# Keratin-water-NMF interaction as a three layer model in the human stratum corneum using *in vivo* confocal Raman microscopy

**DOI:** 10.1038/s41598-017-16202-x

**Published:** 2017-11-21

**Authors:** ChunSik Choe, Johannes Schleusener, Jürgen Lademann, Maxim E. Darvin

**Affiliations:** 1 Charité – Universitätsmedizin Berlin, corporate member of Freie Universität Berlin, Humboldt-Universität zu Berlin, and Berlin Institute of Health, Department of Dermatology, Venerology and Allergology, Center of Experimental and Applied Cutaneous Physiology, Charitéplatz 1, 10117 Berlin, Germany; 2 Kim Il Sung University, Ryongnam-Dong, Taesong District, Pyongyang, Democratic People’s Republic of Korea

## Abstract

The secondary and tertiary structure of keratin and natural moisturizing factor (NMF) are of great importance regarding the water regulating functions in the stratum corneum (SC). In this *in vivo* study, the depth-dependent keratin conformation and its relationship to the hydrogen bonding states of water and its content in the SC, are investigated using confocal Raman microscopy. Based on the obtained depth-profiles for the *β*-sheet/*α*-helix ratio, the stability of disulphide bonds, the amount of cysteine forming disulphide bonds, the buried/exposed tyrosine and the folding/unfolding states of keratin, a “three layer model” of the SC, regarding the keratin-water-NMF interaction is proposed. At the uppermost layers (30–0% SC depth), the keratin filaments are highly folded, entailing limited water binding sites, and NMF is mostly responsible for binding water. At the intermediate layers (70–30% SC depth), the keratin filaments are unfolded, have the most water binding sites and are prone to swelling. At the bottom layers (100–80% SC depth), the water binding sites are already occupied with water and cannot swell substantially. The hydrogen bonding states of water molecules can only be explained by considering both, the molecular structure of keratin and the contribution of NMF as a holistic system.

## Introduction

Keratin is one of the abundant proteins found in the mammalian epidermis. Corneocytes of the stratum corneum (SC), the horny cells of human epidermis, are continuously proliferating from the stratum granulosum (SG) towards the skin surface^1,2^, contain a lot of the fibrous keratin^3^ which is embedded in a water-lipid matrix^4^, and is almost homogenously distributed throughout the SC of healthy skin^5^. However, this can be different in the diseased skin^6^. The SC has been recognised as the skin barrier, which prevents exogenous substances from penetrating into the skin and also for water evaporation out of the body^7–9^. The barrier function of the SC is primarily maintained by the lateral packing order of intercellular lipids (ICL)^10^, which is distributed non-homogeneously in the SC^11,12^ and can vary age-dependently^13^. Keratin filaments are recognised as a major factor for maintaining the mechanical skeleton of the corneocytes and, together with corneodesmosomes, the durability of the SC. They participate in the regulation of the water diffusion process, and the occlusion-based swelling effect^14–18^. Meanwhile, the distribution of bound/unbound water in the SC and the trans-epidermal water loss have been important issues in dermatology and cosmetology^15,17–20^.

**Figure 1:**
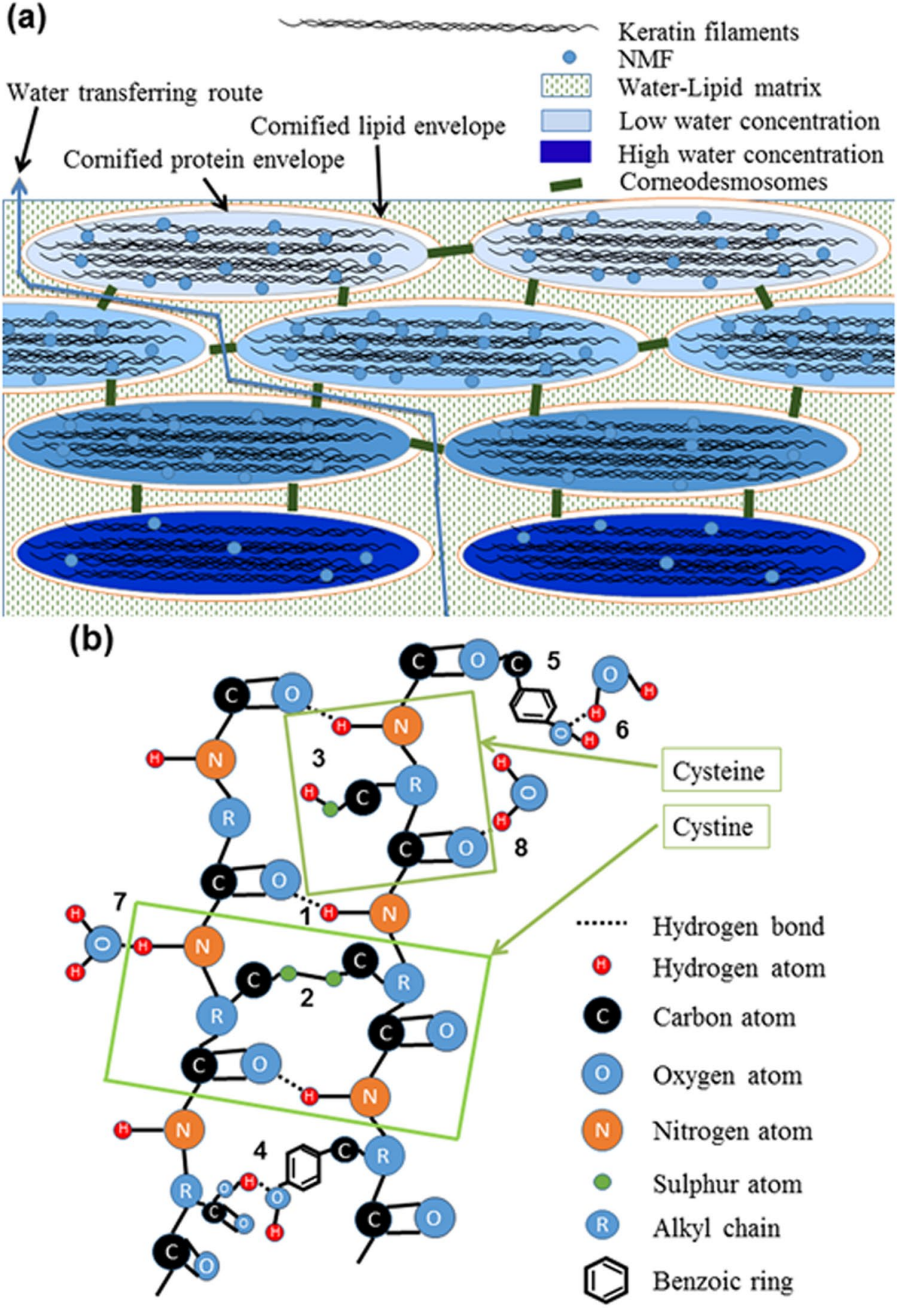
Schematic structure of the SC and keratin filament. (**a**) The schematic structure of the SC, which consists of corneocytes embedded in a water-lipid intercellular matrix. The corneocytes consist of keratin fibres, NMF and water. (**b**) The structure of a keratin filament and the types of side-chain reactions. 1: The hydrogen bonds between N–H and C=O groups (corresponding to the Amide I Raman band around 1655 cm^−1^), 2: disulphide bonds between two cysteine side-chains (corresponding to the Raman band around 491 cm^−1^ and 546 cm^−1^), 3: free cysteine side-chain that does not form disulphide bonds (corresponding to the Raman band around 700 cm^−1^), 4: the buried- and 5: exposed tyrosine side-chains in keratin chains (corresponding to the Raman bands around 830 cm^−1^ and 850 cm^−1^), 6, 7, 8: the hydrogen bound water molecules with the keratin chains, also showing the water-binding sites of keratin chains.

Proteins such as keratin are described on different levels of biomolecular structure. The primary structure is related to the order of amino acids in keratin chains. The secondary structure characterises the coiled, *β*-sheet and *β*-turns and random coil structures of proteins by the hydrogen bonds between N–H and C=O bonds. The tertiary structure of keratin describes the folding/unfolding structure of proteins, caused by the interaction of side-chains of keratin chains, e.g. disulphide bonds of cysteine, hydrogen bonds of tyrosine and other possible electrostatic forces between the side-chains of protein. Since most of the water is present in the corneocytes, rather than in the lamellas of ICL^21^, and the corneocytes largely consist of keratin filaments and hygroscopic natural moisturizing factor (NMF) (Fig. 1a), the secondary and tertiary structures of keratin are important for the water regulation in the SC. The structure of keratin depends on its extensive intra- and inter-molecular interactions between side-chains of keratin filaments. One of these interactions is the formation of disulphide bonds by sulphur-sulphur crosslinking between two cysteine molecules in the keratin framework^22–24^ (Fig. 1b). According to the amount of their sulphur contents, which are only present in cysteine and methionine amino acids of keratins, keratins are categorised into “hard”-keratin (higher sulphur contents) and “soft”-keratin (lower sulphur contents). The keratin in the SC belongs to the category of “soft”-keratin^22,23^.

Vyumvuhore *et al*.^19^ highlighted the co-relationship between the conformations of the keratin filaments and the bound/free water in the SC by changing the relative humidity. In contrast, Choe *et al*.^25^ showed uncorrelated profiles of the keratin’s folding/unfolding properties and the hydrogen bonding state of water, i.e. strongly bound/weakly bound water in the SC as a function of SC depth. In relation to hydrogen bonding states of water, it is also noteworthy to consider that NMF is hygroscopic material, and that ICL maintains the main water diffusion pathways in the SC^17,18^.

Confocal Raman microscopy (CRM) has been introduced for non-invasive determination of the chemico-physical properties of the SC *in vivo* and *ex vivo*, with sufficient spatial resolution^25,26^.

In this study, the depth-dependent molecular structures of keratin, i.e. the secondary and tertiary structures, are thoroughly investigated using CRM in the human SC *in vivo*, mainly by analysing keratin-related Raman bands. The study will also contribute to the understanding of the biophysical properties of corneocytes, as the system of keratin-water-NMF interaction, microscopically in the whole human SC *in vivo*.

## Results

### The Raman indicators for keratin conformation in the human SC

**Figure 2:**
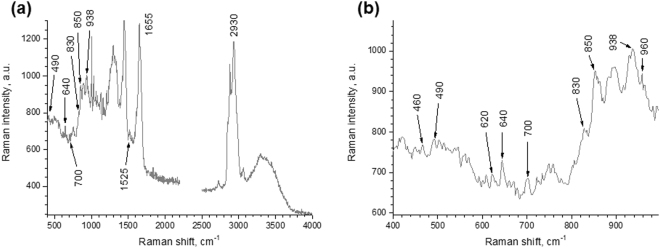
Raman spectra of human SC *in vivo*. (**a**) The Raman spectrum of the forearm skin at the depth of 8 µm (male, age 27). (**b**) Detailed depiction of the same spectrum in the 400–1000 cm^−1^ range. The SC consists of keratin- and NMF-rich corneocytes, a lipid matrix in the intercellular space, water and carotenoids. Table 1 shows the assignments of the Raman bands.

Figure 2 shows the Raman spectrum measured at the forearm skin in the depth of 8 µm.

**Table 1:** The assignments of the Raman bands of the SC *in vivo* (as presented in Fig. 2), according to^22,23,34,39,64^.

Wavenumber (cm^−1^)	Assignment	Origin of the Raman peak	The depth-dependent behaviour in human SC
491	ν(SS), [*gauche*-*gauche*-*gauche*]	keratin	Appears strongly at the upper layers of the SC
545	ν(SS), [*gauche*-*gauche*-*trans*]	keratin	Appears as a broad band from the intermediate layers,
620	ν(CS)	keratin	Weak peak at the upper layers of the SC
640	ν(CS), [*gauche*]	keratin	Weak sharp peak in all depths
700	ν(CS), [*gauche*]	keratin	Weak peaks in all depths
702		Cholesterol, cholesterol ester	Weak in all depths
745	ρ(CH_2_) in-plane	keratin	Broad bands from intermediate layers of the SC
830	δ(CCH) aromatic	keratin (tyrosine)	Weak and shoulder peak in the upper layers and independent peak from the intermediate layers
850	δ(CCH) aromatic	keratin (tyrosine)	Strong in all depths
880–890	ν(C–C) skeleton vibration from long hydrocarbon chains or ρ(CH_2_), γ(CH_3_)	lipids (free fatty acid or ceramide) and ρ(CH_2_) (keratin)	Strong broad peak in all depths
932	ν(C–C) *α*-helix conformation	keratin	Strong peak in all depths
960	ν(C–C) *β*-sheet conformation	keratin	Weak shoulder peaks
1003	ν(CC) aromatic	phenylalanine of keratin and urea (NMF)	Strong in all depths
1032	ν(CC) skeletal, *cis*- conformation	keratin	Medium in all depths
1062	ν(CC) skeletal, *trans*-conformation	lipids	Medium in all depths
1080	ν(CC) skeletal, *gauche*-conformation	lipids	Weak
1130	ν(CC) skeletal, *trans*-conformation	lipids	Strong in all depths
1156	ν(CC) skeletal vibration	lipids and carotenoids	Medium in the upper layers of the SC
1180	ν(CC) skeletal vibration	lipids	Weak in all depths
1206–1210	ν(CC) skeletal vibration	lipids and small contribution of keratin	Weak in all depths
1298	δ(CH_2_) deformation	lipids	Strong and sharp in all depths
1320–1342	δ(CH_2_) deformation	lipids	Shoulder
1450	δ(CH_2_) scissoring	lipids (sensitive to hexagonal and liquid like phase of lipids) and keratin	Strong in all depths
1523	ν(CC) skeletal vibration	carotenoids	Weakly appears in the upper layer
1616	ν(CC) skeletal vibration	lipids	Shoulder peak
1655	amide I (C=O) vibration, *α*-helix conformation	keratin and very little contribution of lipids	Strong in all depths
2850	ν(C–H) symmetric vibration	lipids	Strong in all depths
2880	ν(C–H) asymmetric vibration	Mostly lipids and the small contribution of keratin	Strong in all depths
2930	ν(CH_3_) symmetric stretching	Mostly keratin and the small contribution of lipids	Strong in all depths
2980	ν(CH_3_) asymmetric stretching	keratin	Shoulder of 2930 cm^−1^ peak
3063	ν(=CH) stretching	keratin	Strong in all depths
3100–3800	O–H vibration	water	Strong in all depths

ν denotes stretching vibrations, ρ wagging vibration modes and δ bending vibration modes.

### Depth-dependent changes of the secondary structure of keratin determined by the *β*-sheet/*α*-helix ratio

There are two forms of secondary structure of keratin, *α*-helix and *β*-sheet. The *α*-helix keratin, a highly stable form of keratin, has a coiled-coil structure and less exposed side-chains, which does not efficiently interact with water and other biomolecules. The coiled-coil structure is a unique characteristic of *α*-helix keratin filaments because of crosslinking formed by disulphide bonds or the hydrogen bonds of tyrosine side-chains of keratin^27^. The *β*-sheet keratin is softer than the *α*-helix and has a relatively large number of exposed side-chains between the sheets. Thus, water molecules can easily intercalate between the sheets of *β*-sheet keratin and bind with hydrogen bonds.

The two Raman bands at 938 cm^−1^ and 960 cm^−1^ originating from the C–C skeleton vibration, serve as indicators for *α*-helix and *β*-sheet keratin (Table 1). The Raman band at 938 cm^−1^, which appears strongly for all SC depths, corresponds to *α*-helix, while the band at 960 cm^−1^, which is the shoulder of the peak at 938 cm^−1^, corresponds to *β*-sheet^15,28–31^. The ratio of the 960 cm^−1^/938 cm^−1^ (area under the curve, AUC, 952–966 cm^−1^/924–946 cm^−1^) is calculated and is considered an indicator of the *β*-sheet/*α*-helix ratio of keratin.

**Figure 3:**
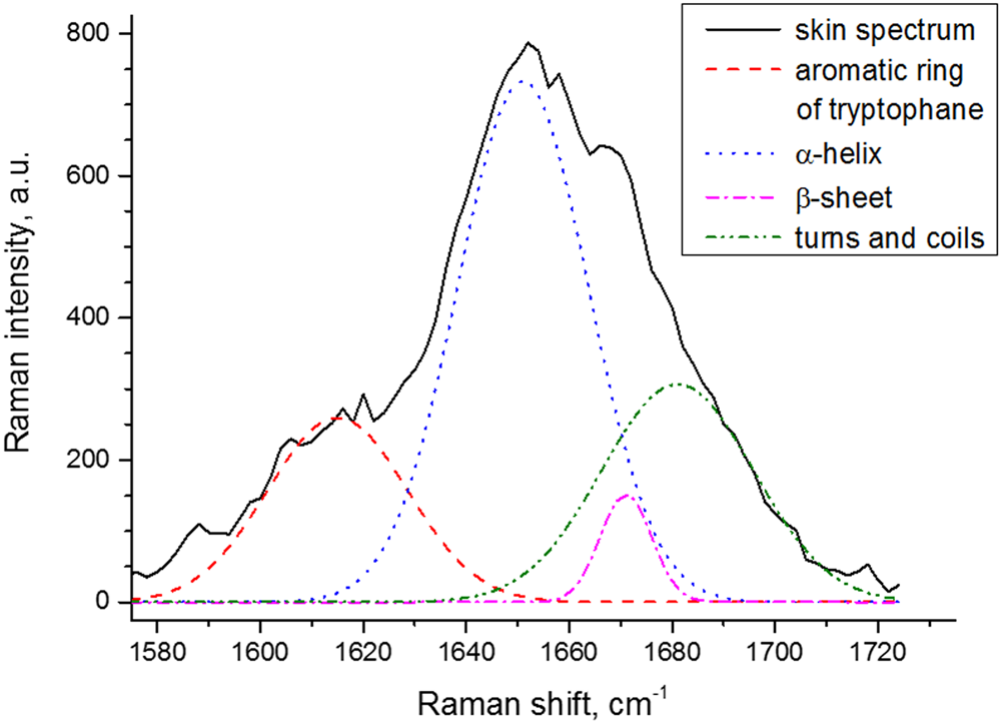
Deconvolution of the Amide I band of human SC *in vivo*. 4 Gaussian bands centred at 1617 ± 7 cm^−1^, 1655 ± 5 cm^−1^, 1670 ± 5 cm^−1^ and 1685 ± 5 cm^−1^ with the corresponding FWHM equal to 23 ± 10 cm^−1^, 30 ± 6 cm^−1^, 15 ± 7 cm^−1^ and 37 ± 7 cm^−1^, are used, respectively. The spectrum is obtained *in vivo* at 8 µm depth from the forearm of a male volunteer (age 27).

The shape of the Amide I band of keratin (1580–1720 cm^−1^) is also considered to be a useful criterion to obtain information about secondary structures of keratin. The keratins of the SC are normally arranged in *α*-helical stable conformation^24^, as shown in Fig. 3 by the peak centred at 1655 cm^−1^, but there is also a small amount of *β*-sheet conformation^23,32^, visible from the shoulder peak at around 1670 cm^−1^. In order to estimate the *β*-sheet/*α*-helix ratio, a Gaussian deconvolution method is applied for the range 1580–1720 cm^−1^ using 4 Gaussian bands^19^ (Fig. 3).

This adapted the constrained non-linear optimization method to minimise the fitting residual errors between experimental and modelled spectra^33^. In order to obtain reproducible and biochemically reasonable results, the full widths at half maximum (FWHM) of 4 Gaussian bands were allowed to change within a limited range of 10 cm^−1^. The Gaussian band, centred at 1617 cm^−1^, is assigned to aromatic vibration of phenylalanine, tyrosine and tryptophan^34,35^. The band at 1655 cm^−1^ is related to *α*-helix, the band at 1670 cm^−1^ corresponds to the *β*-sheet conformation and the band at 1685 cm^−1^ is related to turns and random coils of keratin^19,34,36^. The *β*-sheet and turns and random coils of keratin represent the most disordered structures, whose hydrogen bonds within its chains do not interact with the side-chains of keratin (exposed state), entailing increased possibility to bind water molecules.

**Figure 4:**
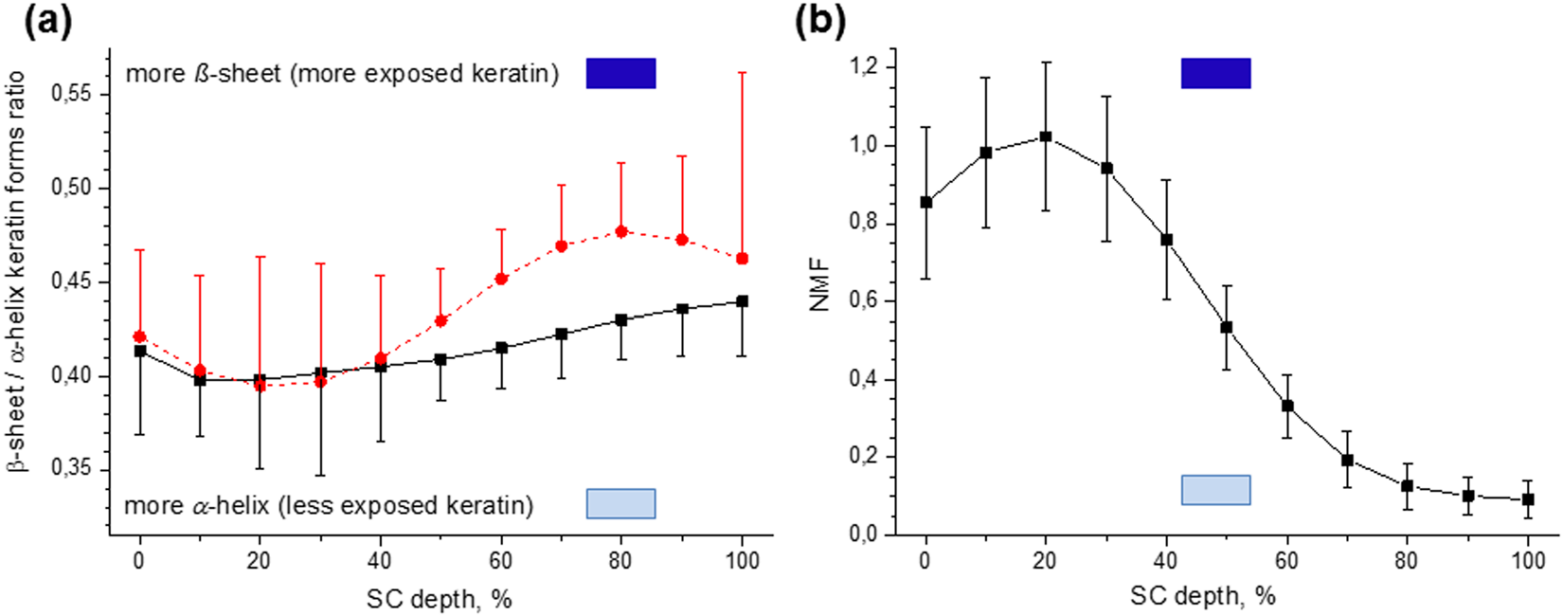
The depth profile of the *β*-sheet/*α*-helix keratin and NMF concentration in the human SC *in vivo*. The *β*-sheet/*α*-helix is determined by the 960 cm^−1^/938 cm^−1^ ratio (squares, black) and the (*β*-sheet + turns and random coils)/*α*-helix keratin forms ratio determined by the (1670 cm^−1^ + 1685 cm^−1^)/1655 cm^−1^ AUGCs ratio of the deconvoluted amide I band (cycles, red dotted) (**a**) and the NMF depth profile (**b**). Mean ± standard deviation for 11 volunteers. / lower/higher possibility to bind water molecules.

The *β*-sheet/*α*-helix ratio calculated by the 960 cm^−1^/938 cm^−1^ ratio decreases as a trend (*p* < 0.1) from the 100–40% SC depth, but does not change significantly at 40–10% SC depths (*p* > 0.1). At the skin surface, although its average is slightly larger compared to the 10% SC depth, no significant difference was found (*p* = 0.17) (Fig. 4a, black line). The depth-dependent (*β*-sheet + turns and random coils)/*α*-helix ratio, calculated by the (1670 cm^−1^ + 1685 cm^−1^)/1655 cm^−1^ ratio of area under the Gaussian curves (AUGCs), which also decreases from 70% to 40% SC depth (*p* < 0.05), remains constant at the 40–10% SC depth and slightly increases from 10% SC depth to the skin surface (*p* < 0.1) (Fig. 4a, red dotted line). Summarizing the results shown in Fig. 4a, keratin filaments transform from the less stable and more exposed *β*-sheet form to the highly stable and less exposed *α*-helix form from the bottom towards the upper SC layers. The *α*-helix keratin form remains constant at 40–10% SC depth, providing less amount of vacant positions for water binding in comparison to other SC depths. At the skin surface, *β*-sheet keratin slightly increases as a trend (Fig. 4a) (p < 0.1).

Figure 4b shows the depth profile of the NMF molecules in the SC with a prominent maximum at 20% SC depth.

### The depth-dependent tertiary structure of keratin determined by the strength of disulphide bonds and the amount of C–S groups that do not take part in disulphide bonds

**Figure 5:**
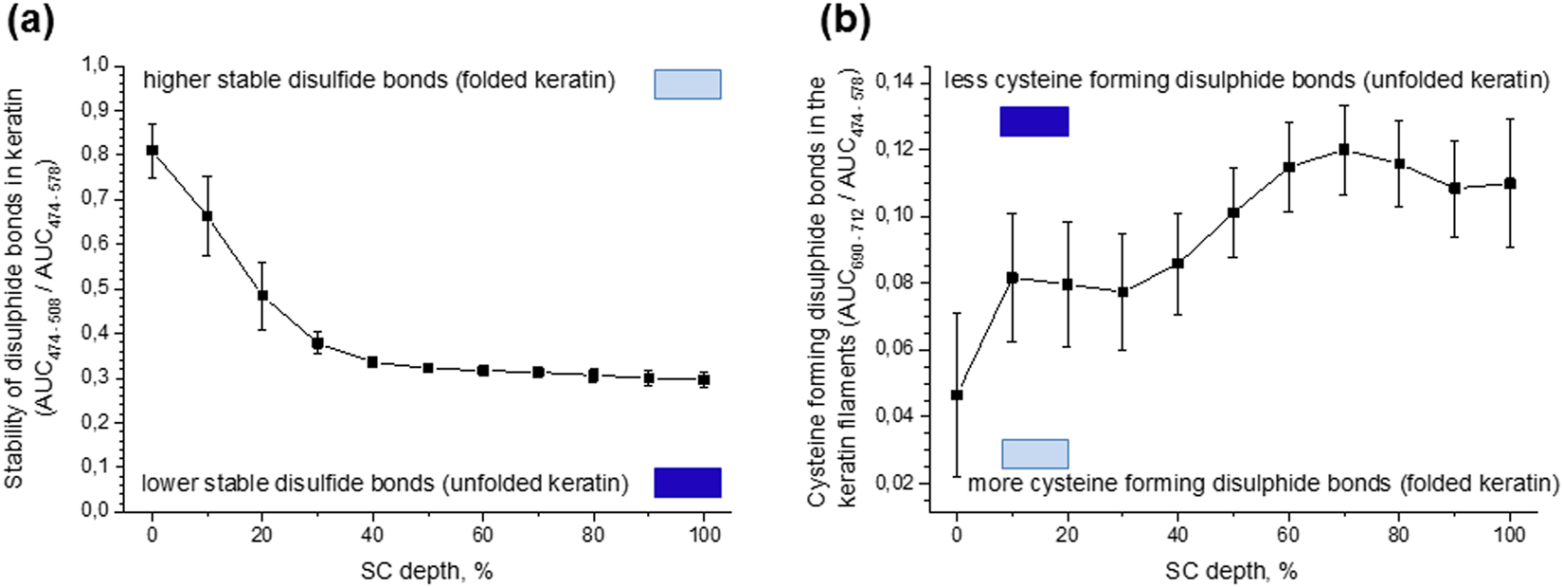
The depth-dependent stability of disulphide bonds and the amount of cysteine in keratin chains in the human SC *in vivo*. The stability in keratin is determined by the ratio of *gauche*-*gauche*-*gauche* conformation (474–508 cm^−1^ AUC) to total disulphide bonds *gauche-gauche-gauche* + *gauche-gauche-trans* + *trans-gauche-trans* (474–578 cm^−1^ AUC) (**a**). The amount of cysteine in keratin chains which form disulphide bonds determined by the C–S (690–712 cm^−1^ AUC) / S–S (474–578 cm^−1^ AUC) ratio (**b**). Mean ± standard deviation is shown for 11 volunteers. / lower/higher possibility to bind water molecules.

Disulphide bonds (–S–S–), which can only be formed between two cysteine amino acids in the keratin structure (Fig. 1b), are important for the stabilization of the higher-order structure of keratin. Disulphide bonds result from the fusion of two cysteine molecules in keratin filaments and the oxidation of sulfhydryl groups that create cystine^22^. In order to measure the stability of the disulphide bonds in keratin, the conformational order of disulphide bonds is calculated in the 474–578 cm^−1^ range, which originates from vibrations of –C–S–S–C– bonds^22^. Here, three Raman bands appear: the band around 491 cm^−1^ is assigned to the *gauche*–*gauche*–*gauche* conformation^22^ and the bands around 525 cm^−1^ and 546 cm^−1^ correspond to the *gauche*-*gauche*-*trans* and *trans*-*gauche*-*trans* conformations of disulphide bonds, respectively^22,23^. Among these three conformations of disulphide bonds, the *gauche*-*gauche*-*gauche* conformation is most stable. The ratio of the *gauche*-*gauche*-*gauche* conformation to the total disulphide bonds (*gauche*-*gauche*-*gauche* + *gauche*-*gauche*-*trans* + *trans*-*gauche*-*trans*), which is introduced here, is considered a criterion for the strength of disulphide bonds in keratin, entailing a more stable folding structure of keratin. A higher/lower ratio represents a higher/lower stability or strength of disulphide bonds, which shows the increased folding/unfolding state of keratin with lower/higher possibility to bind water molecules. Figure 5a shows this ratio. At the 100–50% SC depth, the *gauche*–*gauche*–*gauche* conformation remains almost constant (≈0.3, *p* > 0.1) and is significantly lower than in the upper layers (50–0% SC depth) (*p* < 0.01). From 30% SC depth towards the skin surface, this ratio increases highly significant (*p* < 0.01). At the surface of the skin it indicates that ≈0.8 of total disulphide bonds are in the *gauche*–*gauche*–*gauche* conformation, i.e. in the highly stable form of disulphide bonds.

The AUC of the 474–578 cm^−1^ range represents the total amount of disulphide bonds in keratin. The Raman peak area around 700 cm^−1^ (690–712 cm^−1^) represents the C–S vibrations of the cysteine-related substances, e.g. acetyl-cysteine and methyl-cysteine^37,38^. Specifically the peak around 700 cm^−1^ corresponds to the Raman signal of N–C–C–S groups which is unique to cysteine^23^ (Fig. 1b). A small contribution of cholesterol to this peak also exists^39^, but its influence is significantly lower than that of cysteine. The close-located Raman peak around 640 cm^−1^ does not appear in the cysteine molecules and might be related to methionine, because the H–C–S– group, which is a part of methionine, has Raman bands in the 630–670 cm^−1^ range^23^. Methionine cannot form disulphide bonds, therefore the peaks around 620 cm^−1^ and 640 cm^−1^ related to C–S vibration of methionine, are excluded from the calculation of the total cysteine/disulphide linkages, i.e. the C–S/S–S ratio.

Not all cysteine molecules form a disulphide linkage in keratin filaments (Fig. 1b). Thus, the ratio between the amounts of the C–S groups (690–712 cm^−1^ AUC), characterizing the total cysteine concentration and the amount of cysteine molecules forming the disulphide bonds S–S (474–578 cm^−1^ AUC) in keratin filaments, could be an indicator of the amount of cysteine molecules taking part in building the disulphide bonds in keratin chains. A larger ratio indicates that less cysteine takes part in the formation of disulphide bonds in keratin chains. Thus, the interactions between the side-chains of cysteine are weak, leading to the conclusion that the keratin chains are less folded and are potentially able to bind water molecules. In contrast, a lower ratio indicates a higher amount of cysteine taking part in building the disulphide bonds in keratin chains, thus increasing folding of the keratin chains and decreasing ability of keratin to bind water molecules.

Figure 5b shows the ratio of the total amount of cysteine to the cysteine forming the disulphide linkages in keratin chains. From the bottom to 70% SC depth, this ratio slightly increases (*p* < 0.05) to a maximum (≈0.12) at 70% SC depth and then decreases gradually (*p* < 0.01) towards the intermediate layers of the SC (70–40% SC depth), indicating that the amount of “disulphide bonded” cysteine in keratin chains increases. From 40–10% SC depth, this value remains constant around 0.08. At the superficial layers of the SC (10–0% SC depth) the ratio decreases with a steeper slope (*p* < 0.01), indicating an even higher amount of disulphide linkages formed between cysteine in the keratin fibrils. These findings indicate that the number of the disulphide bonds is larger in the upper layers (30–0% SC depth) than in the deeper layers of the SC (100–40% SC depth), and that disulphide bonds are in a most stable state in the upper layers (Fig. 5a). Thus, the keratin filaments are most folded in the upper SC layers (30–0% SC depth) with very limited ability to bind water molecules in comparison to remaining SC depths (100–40% SC depth).

### The depth-dependent tertiary structure of keratin determined by the ratio of buried/exposed tyrosine in the keratin framework

The position of the aromatic ring in the side-chains of the amino acid tyrosine is important to evaluate keratin’s side-chain interactions. There are two configurations of tyrosine, i.e. buried and exposed aromatic rings of tyrosine in keratin side-chains (Fig. 1b). The buried tyrosine forms the hydrogen bonds with the side-chains of keratin, i.e. carbonyl group of other amino acids and not with water molecules, resulting in increased folding of the keratin chains. The exposed tyrosine indicates the unfolded states, resulting in more hydrogen bonds with water and other biomolecules outside the side-chains of keratin^19^. The Raman doublets near 850 cm^−1^ and 830 cm^−1^ correspond to the ring breathing mode of the tyrosine side-chain^22,23^, and are sensitive to the position of tyrosine in the keratin framework (Table 1). The 850 cm^−1^/830 cm^−1^ peak ratio is considered to indicate the exposed/buried ratio of tyrosine side-chains^19,40^. In this study, the inverse ratio of AUCs, i.e. of the peaks around 830 cm^−1^ (816–838 cm^−1^ AUC) and 850 cm^−1^ (838–874 cm^−1^ AUC), was calculated, because the 830 cm^−1^ peak intensity is normally smaller than the 850 cm^−1^ peak intensity, entailing increased standard deviation if this value were placed in the denominator. A lower 830 cm^−1^/850 cm^−1^ ratio indicates more exposed side-chains of tyrosine, indicating more unfolded states of keratin, i.e. the possibility to bind the water molecules, while a larger ratio indicates more buried side-chains of tyrosine, i.e. more folded states of keratin with limited possibility to bind water molecules.

**Figure 6:**
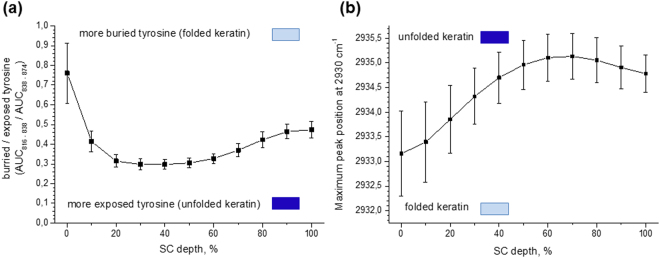
The depth-dependent tertiary structure of keratin in the human SC *in vivo*. The ratio of buried/exposed tyrosine determined by the 830 cm^−1^/850 cm^−1^ ratio (**a**), and the folding/unfolding state of keratin determined by the Gaussian peak position at 2930 cm^−1^ (**b**). Mean ± standard deviation for 11 volunteers. / lower/higher possibility to bind water molecules.

Figure 6a shows the depth profile of the buried/exposed tyrosine ratio in keratin chains. From 100% to 50–20% SC depth, the ratio of buried/exposed tyrosine decreases to minimal values (≈0.3%) (*p* < 0.01), indicating a higher amount of exposed tyrosine in these depths, i.e., more vacant positions for water binding. At the upper layers (20–0% SC depth), the value strongly increases (*p* < 0.01), indicating that the tyrosine molecules transform to the buried state, which means that the side-chain of tyrosine transforms into more buried states, i.e. more hydrogen bonded linkages with other groups turning keratin filaments into the more folded state.

### The depth-dependent tertiary structure of keratin determined by the folding/unfolding states of CH_3_ residues in keratin chains

The Raman peak at 2930 cm^−1^ originates from aliphatic CH_3_ vibration of keratin chains^24,33^ and its position is sensitive to the exposed states of CH_3_ side-chains of keratin, i.e., keratin´s folding/unfolding states^41^. A lower position of the 2930 cm^−1^ peak corresponds to the keratin folding state and a higher position represents the unfolding keratin state, which is associated with the enhanced exposure of CH_3_ side-chains to the surrounding water molecules^24,42^. In order to determine 2930 cm^−1^ peak position, the deconvolution procedure in the HWN region was performed^43^.

As shown in Fig. 6b, maximal unfolding of the keratin filaments occurs at 70–60% SC depth and unfolded keratin decreases from these layers towards the surface (60–0% SC depth, *p* < 0.01 for adjacent depths). Thus, close to the skin surface at 20–0% SC depth, keratin lies in the most folded state. Meanwhile, at the boundary of the SC and the SG, keratin lies in a more folded state than at the 60% SC depth (*p* < 0.01 between 60% and 100% SC depth).

### The depth-dependent water profile and hydrogen bonding state of water

**Figure 7:**
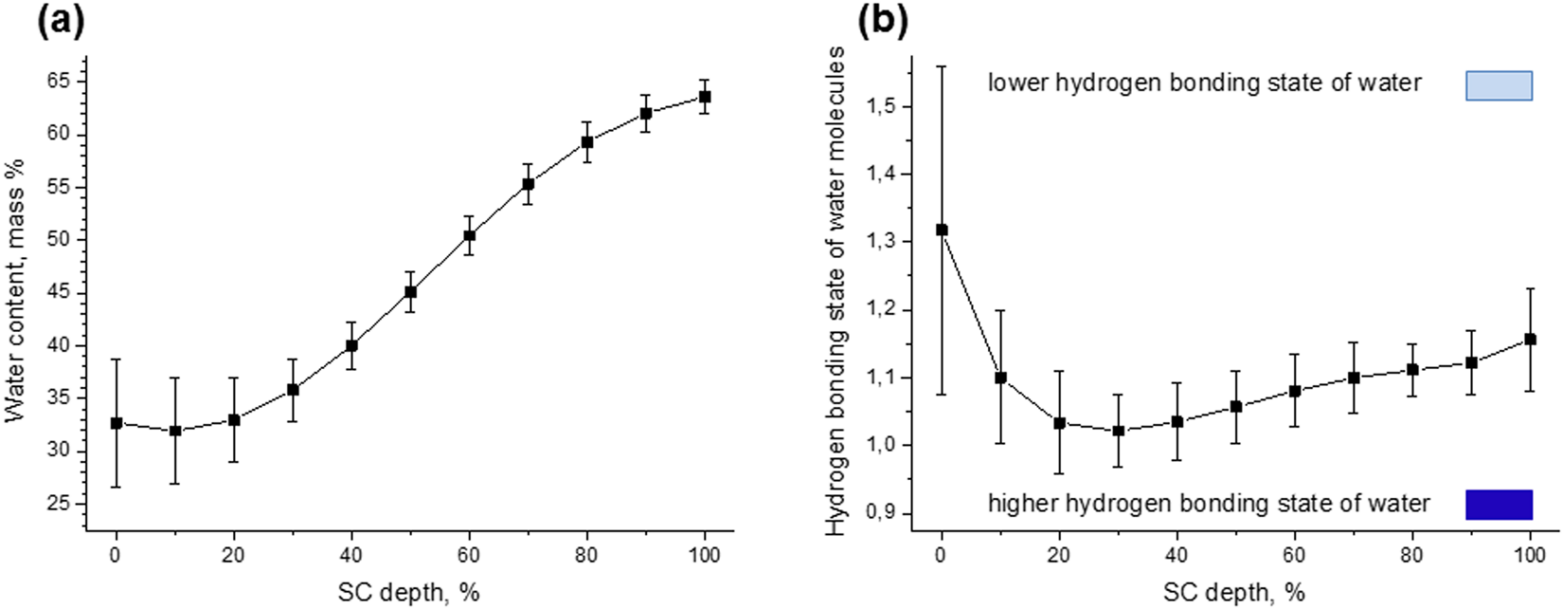
Water concentration and hydrogen bonding state of water in the human SC *in vivo*. Depth profiles of total water content in the SC in mass percent (**a**) and the hydrogen bonding state of water molecules determined by the weakly bound/strongly bound water molecules ratio (**b**). Mean ± standard deviation for 11 volunteers. / lower/higher possibility to bind water molecules.

Figure 7a shows the depth profile of the water concentration in the SC. The water mass content in the SC decreases from the bottom (100% SC depth) towards the surface (10–0% SC depth). The highest gradient occurs at 60–40% SC depth.

Figure 7b shows the depth profile of the hydrogen bonding state of the water molecules determined as a ratio of weakly bound to strongly bound water molecule types, determined according to the recently developed algorithm^25^. This ratio decreases from the bottom towards the upper layers (100–40%) of the SC (*p* < 0.05), and reaches a minimum value (highest hydrogen bonding state of water molecules) at 40–20% SC depth. Then, it significantly increases towards a surface at 10–0% SC depth (*p* < 0.05), showing a decrease of hydrogen bonding states.

## Discussion

The results of the present study can be categorised largely into two groups. One is related to the secondary (Fig. 4a) and the other is related to the tertiary structure, i.e. folding/unfolding state of keratin in the SC (Figs 5 and 6).

### The secondary structure of keratin

Figure 4a (black line) shows an increase of *α*-helix structure by approx. 0.03 with very large standard deviations at 80–20% SC depth, which is determined by analysis of the C–C skeleton vibration Raman bands ratio 938 cm^−1^/960 cm^−1^. This finding suggests that, although more quantitative analysis is necessary, the transformation from *β*-sheet to *α*-helix can be determined as a trend (*p* = 0.07 at 80–20% SC depth).

As shown in Fig. 4a (red dotted line) by analysing the Amide I peak (1580–1720 cm^−1^), the secondary structure of keratin does not change at the deep located layers (100–80% SC depth), the *α*-helix structure increase at 70–40% SC depth and at the upper layers (40–10% SC depth), the ratio remains constant showing more *α*-helical keratin form. At 10–0% SC depth, the amount of *β*-sheet keratin increases again as a trend.

Figure 4a shows that the analyses of the Amide I peak (1580–1720 cm^−1^) are preferable for the determination of the *α*-helix and *β*-sheet secondary structure of keratin, which is confirmed by the lower standard deviation.

### The tertiary structures of keratin

The stability of disulphide bonds (Fig. 5a) remains constant at the deeper SC layers (100–50% SC depth) and substantially increases towards the skin surface (40–0% SC depth). Close to the skin surface, the stability of disulphide bonds in the keratin filaments reaches its maximum of approx. 0.8. The amount of cysteine involved in the formation of disulphide bonds increases from 70–40% SC depth and reaches a plateau at 40–10% SC depth (Fig. 5b). Close to the surface (10–0% SC depth), a significant increase of disulphide linkages formed between cysteine is observed. These tendencies of disulphide bonds show that the interactions between keratin side-chains monotonically increase to more folded states from the bottom towards the surface of the SC. The obtained profiles explain complementary that *α*-helix keratin, which is formed by disulphide bonds, is more pronounced in the 40–0% SC depth compared to the 100–50% SC depth, which is in accordance to results presented in Fig. 4a.

Figure 6a shows tendencies, that exposed tyrosine is most prevalent at 60–20% SC depth (ratio approx. 0.3), while in the upper layers of the SC (20–0% SC depth) and in the deep-located layers (100–70% SC depth) the buried tyrosine is increased. This is obvious for the surface, where the ratio reaches almost 0.8. The folding/unfolding state of keratin determined by the CH_3_ vibration, reaches a maximum unfolding state at 80–50% SC depth (Fig. 6b). The increased folding state is observed close to the surface (20–0% SC depth). At the uppermost layers of the SC (20–0% SC depth), the results shown in Fig. 6a,b correlate, indicating that buried tyrosine and folded keratin are increased. The correlation between the keratin conformations, e.g. the maximum peak position at 2930 cm^−1^ (CH_3_ vibrations), and the buried/exposed tyrosine with changing atmospheric relative humidity has been shown by Vyumvuhore *et al*.^19^ *ex vivo*. In the present study, the buried/exposed tyrosine and CH_3_ vibrations are not correlated at 40–20% SC depth. Figure 6b shows the decrease of the maximum position of the 2930 cm^−1^ band, while the buried/exposed ratio remains constant (Fig. 6a). Also, discrepancies appear between Figs 5 and 6a, which might be due to the representation of different aspects of the side-chain interactions and conformations. An explanation for the limited correlation could be the highly complex organization of keratins, which exhibit interactions between many side-chains. While some side-chain interactions, e.g. those of tyrosine, disulphide bonds, CH_3_, –N–H and C=O establish the highly ordered structure, these interactions do not necessarily take place coincidently and have identical depth profiles in the skin.

### The relationship between the depth profile of water and the secondary and tertiary structures of keratin in the SC

Regarding the secondary structure, the keratin filaments are known to undergo conformational changes from *α*-helix to *β*-sheet when increasing the relative humidity^19,27^.

As shown in Fig. 7a, the concentration of water decreases rapidly towards the surface of the SC, which is equivalent to a decrease of the micro-environmental relative humidity. However, the keratin conformation from *β*-sheet to *α*-helix (Fig. 4a) do not match the water concentration gradient and show only a correlation as a trend. Figure 4a indicates a small decrease of *β*-sheet ≈0.03 with a large standard deviation (black line) and ≈0.08 (red dotted line) from 80–20% SC depth in relation to the water contents (≈26% decrease of water mass %).

Furthermore, regarding the tertiary keratin structure, Fig. 5a indicates the increase of the keratin folding states at 30–0% SC depth with increased stability of disulphide bonds. At the 100–30% SC depth, the folding states of keratin do not change, while the water concentration decreases. This contradiction is also apparent in Fig. 5b, showing constant values at 30–10% SC depth, characterizing the folded state of keratin in comparison to other SC depths. The contradiction in Fig. 6 is even more evident with regard to the depth profile of water. The water concentration decreases monotonically from 100–20% SC depth while the exposed tyrosine increases from 100–20% SC depth (Fig. 6a), leading to the conclusion that the unfolding keratin increases and has a maximum at 80–50% SC depth (Fig. 6b). These contradictions could be explained by the influence of keratin, water and NMF as a holistic system, which needs to be investigated in more detail.

### The relationship between keratin, water and NMF in corneocytes and the water in intercellular spaces

Taking into account that almost 80% of the water accumulates in the corneocytes and that keratin filaments fill the corneocytes^44,45^, the relationship between the amount of water, keratin filaments and NMF might play a critical role in water-related response, such as swelling and moisturizing effects^14,19,45,46^. Although the lipids in the intercellular space are considered as a bound-water modulator and the primary diffusion pathway^18,47^, the binding site of water in the lipid’s polar head groups (e.g. ceramides) is small compared to keratin and NMF^47,48^.

Thus, keratin and NMF are the major contributors of thermodynamical water–SC interactions and water firstly binds tightly to the polar sites of keratin, replacing the keratin–keratin bonds to energetically favourable keratin–water bonds^49^. Assuming the corneocytes as NMF-containing keratin sponges^50^, it is debated which contributes more to the water-binding properties between NMF and keratin filaments and how to understand the keratin–water–NMF interaction in corneocytes.

Kasting *et al*.^17,18,49^ suggested that water primarily interacts with keratin more strongly than cellulose or acrylic acid and that the diffusivity value in keratin is >1000-fold lower than in the synthetic polymers. In contrast, Gilard *et al*.^51^ indicated that NMF influences the amount of bound water by using proton nuclear magnetic resonance, which was confirmed by other researchers^20,50,52^.

Speculating that the keratin filaments are the major provider of the water-binding sites, as well as NMF, the number of water-binding sites is mainly determined by the polar sites in side-chains in keratin and its exposed or buried states.

From the knowledge about the keratin conformations obtained in this study (Figs 4–6), we suggest the following depth-dependent mechanisms for keratin–water–NMF interaction in the corneocytes.

### Keratin-water-NMF interaction at 30–0% SC depth

At the uppermost layers (30–0% SC depth), the keratin chains are highly folded and represent predominantly coiled-coil structure (Figs 4–6). Therefore, the keratin filaments have the least binding sites at the uppermost layers, compared to the other SC depths and the number of vacant positions for water binding is minimal in these SC layers. At the uppermost layers (30–0% SC depth), the water mass has its lowest concentration (Fig. 7a), but the NMF concentration is highest (Fig. 4b). Thus, NMF, which bonds water very efficiently, is responsible for the increase of the hydrogen bonding states of water molecules in these depths (Fig. 7b), compensating the low ability of folded keratin to bind water. These findings emphasise the important role of NMF in controlling hydrogen bonding states of water molecules at the uppermost layer of the SC (30–0% SC depth).

### Keratin-water-NMF interaction at the 70–30% SC depth

At the intermediate layers (70–30% SC depth), although keratin chains gradually transform from *β*-sheet into *α*-helix (Fig. 4a), the transforming rate is small compared to the water gradient at these depths (Fig. 7a). It was found that the unfolding states of keratin have a maximum in the intermediate layers (Fig. 6). If we hypothesise that the binding sites of keratin are saturated by the water molecules at 100–80% SC depth and thus the number of water binding sites in keratin chains is equal to the water molecules, the rapid decrease of the amount of water and the increase of exposed polar sites in the side-chains of keratin (created by unfolding keratin at 70–50% SC depth) must create free water-binding sites in keratin chains that are capable to bind water molecules. This hypothesis is supported by the finding that the water turns into more hydrogen bonding states from 70% towards 30% SC depth and reaches the highest hydrogen bonding state at 40–20% SC depth^25^. The reason for the disagreement between the maximum of hydrogen bonding states and the maximum of unfolding states of keratin can be explained by the increased concentration of NMF (Fig. 4b) and highest ICL lateral packing order^11,12^ at 40–20% SC depth.

### Keratin-water-NMF interaction at the 100–80% SC depth

At the bottom layers (100–80% SC depth), the water concentration is highest (Fig. 7a), but the NMF concentration is lowest (Fig. 4b). The folding state of keratin is not maximal, showing a higher value at 80–50% than at 100–80% SC depth (Fig. 6b). The similar tendency is observed in Figs 5b and 6a, where the deep SC layers contain more folded keratin than the close-located 80–50% SC depths.

Therefore, we hypothesise that there are fewer keratin- and NMF-related water binding sites at 100–80% SC depths in comparison with 80–50% SC depths. This causes the increase of unbound and weakly bound water, which is correlated with the weakly bound/strongly bound water ratio (Fig. 7b).

### Three layer model of keratin-water interaction in the SC

Caussin *et al*.^45^ suggested the existence of three different regions in the SC regarding a moisturizing effect in *ex vivo* experiments using cryo-scanning electron microscopy. An upper non-swelling region at the surface of the SC, a swelling region in intermediate layers and a lower non-swelling region at the boundary between SC and SG were suggested, which is supported by the presented *in vivo* results and explained by the microscopic analysis of keratin-water-NMF interaction for the whole SC.

Based on the obtained results of the keratin, water and NMF profiles in the SC, the three layer model of the SC can be explained/specified as follows:

At the uppermost layers (30–0% SC depth), keratin chains are highly folded and stay in a most stable *α*-helix conformation form. Thus, the water cannot intercalate into the keratin chains and there are no vacancies for binding water molecules with keratin. Furthermore, because of the rigid and stable *α*-helix keratin, the corneocytes cannot be swelled mechanically and consequently these layers are characterised as the uppermost non-swelling region. NMF is mostly responsible for binding water in these SC layers.

At the intermediate layers (70–30% SC depth), the keratin chains are more unfolded and therefore, the interaction between side-chains of keratin is weak and the water molecules intercalate into keratin filaments and bind there, entailing the corneocytes to swell. Therefore, this region is denoted as the swelling region.

At the bottom layers (100–80% SC depth), representing the boundary between SC and SG, the keratin chains have fewer vacant binding sites than the intermediate layers due to the more folded keratin than at 70% SC depth, lowest NMF concentration and largest amount of water. Thus, in this SC layer, the incoming water cannot efficiently form hydrogen bonds with the surrounding keratin and NMF, denoting this SC layer as the bottom low-swelling region.

In conclusion, we propose a “three layer model” of the SC by analysing the higher-order structures and the conformations of keratin *in vivo*. Since the hydrogen bonding or bound/unbound states of water molecules cannot be explained by the contribution of NMF alone or the higher-structure of keratin cannot be considered separately, NMF and keratin should be treated as one interacting system, with regard to the keratin-water-NMF interaction in the SC.

## Methods

### Volunteers

11 healthy Caucasian volunteers (9 female, 2 male) aged from 23 to 62 (average 37) years old, who were instructed not to apply any cosmetic substances to the skin for 3 days and not to bath or shower for 4 hours prior to the measurements participated in this study. Ten measurements were taken from within an area of 2 × 2 cm^2^ on the intact forearm skin after an acclimatization time of 20 minutes to the standard room conditions. For each measurement position, spectra from above the skin surface to the depth of 40 µm using 2 µm increments were acquired in the fingerprint region (400–2000 cm^−1^, 5 s acquisition time) and in the high wavenumber region (HWN, 2000–4000 cm^−1^, 1 s acquisition time).

All volunteers had given written informed consent. The experiments were approved by the local ethics committee of the Charité–Universitätsmedizin Berlin and were in accordance with the principles of the declaration of Helsinki as revised in 1996.

### Confocal Raman microscopy (CRM)

A model 3510 SCA confocal Raman Microscope (River Diagnostics, Rotterdam, the Netherlands), suitable for *in vivo* measurements on the skin was used for the experiments. The device uses a 785 nm laser for the analysis in the fingerprint region and a 671 nm laser for analysis in the HWN region. The laser power on the skin surface was 20 mW (1.1 J/cm^2^) and 17 mW (0.2 J/cm^2^), respectively.

The utilised doses of reference light can be considered safe for human skin due to the resulting low local temperature increase (≤2 °C)^53^ and with regard to reference light-induced free radical generation^54,55^. The dose-dependent fluorescence photobleaching in the SC, without influence on the Raman peak intensities^56^, ensures comparable Raman results. A spatial resolution of ≤5 µm and a spectral resolution of 2 cm^−1^ can be achieved. The utilised CRM has been previously described in detail elsewhere^57^.

### Determination of the skin surface and SC thickness

The “Skin tools 2.0” Software by River Diagnostics was used for determination of the skin surface^26^. Thereby, the surface (0 µm or 0%) is determined to be at the position where the keratin depth profile, determined by the Raman peak intensity at 1655 cm^−1^ in the fingerprint region, reaches half of the maximum intensity from outside of the skin. The subsequent depths are corrected according to the applied depth increments during the data acquisition. The SC thickness is determined by the boundary position between the SC and the SG^58^. At this position, the first derivative of the water profile is 0.5, as indicated by Crowther *et al*.^59^. To enable the direct comparison between the volunteers with different SC thickness, the indicated SC depths are normalised to the SC thickness, interpolated to 10% increments of the SC thickness and stated in % SC depth throughout this manuscript. The analysis of the depth-profiles is then performed from the deepest located layers towards the skin surface, according to the direction of transformation of the corneocytes.

### Pre-processing of Raman spectra

In order to calculate the AUC for all Raman bands in the fingerprint region, a linear baseline removal is applied. The 5–10 tangent points on both sides of the peaks are selected and a linear baseline is interpolated to these points by linear least squares regression, which is subtracted from the original Raman spectrum. Principal component analysis (PCA) was applied to reduce the low variability components of the Raman spectral profiles and to calculate the AUC of small intensity Raman bands. The first 4 principal components (PCs) are selected to reconstruct the Raman spectra with reduced variability. From the series of spectra of each measuring point, the spectrum of the selected depth is collected as one sample group. Then, the spectra of samples are PCA-transformed and only the first 4 PCs are used for the reconstruction of the spectra. A detailed description and the effect of error reduction is described elsewhere^12,60^.

In the HWN region, a piecewise-weighted-least squares fitting algorithm was applied in order to maintain the linearity with different gradients in the 2776–2810 cm^−1^ and 3800–3900 cm^−1^ wavenumber regions^25^ without application of the PCA method.

### Analysis of the depth-dependent water profiles in the SC

The depth-dependent profiles of water mass % in the SC were determined by the “Skin tools 2.0” software (River Diagnostics, Rotterdam, The Netherlands), which is based on the adjustment of the OH (3350–3550 cm^−1^ AUC) to the keratin vibration (2910–2965 cm^−1^ AUC) ratio to the real water mass % in the SC determined experimentally^26,61^.

The depth profile of the hydrogen bonding states of water molecules was determined using Gaussian function-based deconvolution of the HWN region (2800–3700 cm^−1^) into 10 sub-bands. The ratio of weakly/strongly bound water was calculated by the ratio of the AUGCs associated to weakly (single donor–single acceptor, 3458 cm^−1^) and strongly (double donor–double acceptor, 3277 cm^−1^) bound water, as explained in detail elsewhere^25^. The deconvolution procedure is performed by using the self-programmed software based on Matlab R2015a (The Mathworks, Inc., Natick, USA).

### Statistical analysis

Statistical evaluation was performed by using Microsoft Excel and the Statistics and Machine learning toolbox of Matlab. In order to test for significant differences of the means of two different depths in the SC, a paired student’s *t*-test was applied^62^. The *t*-test is based on the assumption that all samples are normally distributed. Therefore, a Jarque-Bera test is performed to verify the normal distribution of the data^63^. A *p*-value ≤ 0.01 is considered to be “highly significant”, *p* ≤ 0.05 as “significant”, *p* ≤ 0.1 as “trend”, and *p* > 0.1 is considered as “not significant”. All the values in the figures are plotted with the mean values ±1 standard deviation.

### Data availability

The datasets generated and analysed during the current study are not publicly available due to ethical restrictions.

## References

[1] Sahle FF, Gebre-Mariam T, Dobner B, Wohlrab J, Neubert RH (2015). Skin diseases associated with the depletion of stratum corneum lipids and stratum corneum lipid substitution therapy. Skin pharmacology and physiology.

[2] Sutterlin T, Tsingos E, Bensaci J, Stamatas GN, Grabe N (2017). A 3D self-organizing multicellular epidermis model of barrier formation and hydration with realistic cell morphology based on EPISIM. Scientific reports.

[3] Voloshina OV, Shirshin EA, Lademann J, Fadeev VV, Darvin ME (2017). Fluorescence detection of protein content in house dust: the possible role of keratin. Indoor air.

[4] Elias PM (1979). Localization and composition of lipids in neonatal mouse stratum granulosum and stratum corneum. The Journal of investigative dermatology.

[5] Norlen L (2006). Stratum corneum keratin structure, function and formation - a comprehensive review. Int J Cosmet Sci.

[6] Clausen ML, Slotved HC, Krogfelt KA, Agner T (2016). Tape Stripping Technique for Stratum Corneum ProteinAnalysis. Scientific reports.

[7] Wertz PW (2013). Current understanding of skin biology pertinent to skin penetration: skin biochemistry. Skin pharmacology and physiology.

[8] Bolzinger MA, Briancon S, Pelletier J, Chevalier Y (2012). Penetration of drugs through skin, a complex rate-controlling membrane. Curr. Opin. Colloid Interface Sci..

[9] Johnsen GK, Norlen L, Martinsen OG, Grimnes S (2011). Sorption properties of the human stratum corneum. Skin pharmacology and physiology.

[10] Damien F, Boncheva M (2010). The extent of orthorhombic lipid phases in the stratum corneum determines the barrier efficiency of human skin *in vivo*. The Journal of investigative dermatology.

[11] Doucet J, Potter A, Baltenneck C, Domanov YA (2014). Micron-scale assessment of molecular lipid organization in human stratum corneum using microprobe X-ray diffraction. Journal of lipid research.

[12] Choe C, Lademann J, Darvin ME (2016). A depth-dependent profile of the lipid conformation and lateral packing order of the stratum corneum *in vivo* measured using Raman microscopy. The Analyst.

[13] Choe, C., Schleusener, J., Lademann, J. & Darvin, M. E. Age related depth profiles of human Stratum Corneum barrier-related molecular parameters by confocal Raman microscopy *in vivo*. *Mechanisms of ageing and development*, 10.1016/j.mad.2017.08.011 (2017). 10.1016/j.mad.2017.08.011 28844969

[14] Norlen L, Emilson A, Forslind B (1997). Stratum corneum swelling. Biophysical and computer assisted quantitative assessments. Archives of dermatological research.

[15] Vyumvuhore R (2013). Raman spectroscopy: a tool for biomechanical characterization of Stratum Corneum. Journal of Raman Spectroscopy.

[16] Menon GK, Cleary GW, Lane ME (2012). The structure and function of the stratum corneum. International Journal of Pharmaceutics.

[17] Kasting GB, Barai ND, Wang TF, Nitsche JM (2003). Mobility of water in human stratum corneum. Journal of pharmaceutical sciences.

[18] Kasting GB, Barai ND (2003). Equilibrium water sorption in human stratum corneum. Journal of pharmaceutical sciences.

[19] Vyumvuhore R (2013). Effects of atmospheric relative humidity on Stratum Corneum structure at the molecular level: *ex vivo* Raman spectroscopy analysis. The Analyst.

[20] Boireau-Adamezyk E, Baillet-Guffroy A, Stamatas GN (2014). Mobility of water molecules in the stratum corneum: effects of age and chronic exposure to the environment. The Journal of investigative dermatology.

[21] Silva CL (2007). Stratum corneum hydration: phase transformations and mobility in stratum corneum, extracted lipids and isolated corneocytes. Biochimica et biophysica acta.

[22] Akhtar W, Edwards HG (1997). Fourier-transform Raman spectroscopy of mammalian and avian keratotic biopolymers. Spectrochimica acta. Part A, Molecular and biomolecular spectroscopy.

[23] Edwards HG, Hunt DE, Sibley MG (1998). FT-Raman spectroscopic study of keratotic materials: horn, hoof and tortoiseshell. *Spectrochimica acta*. Part A, Molecular and biomolecular spectroscopy.

[24] Gniadecka M, Faurskov Nielsen O, Christensen DH, Wulf HC (1998). Structure of water, proteins, and lipids in intact human skin, hair, and nail. The Journal of investigative dermatology.

[25] Choe C, Lademann J, Darvin ME (2016). Depth profiles of hydrogen bound water molecule types and their relation to lipid and protein interaction in the human stratum corneum *in vivo*. The Analyst.

[26] Caspers PJ, Lucassen GW, Carter EA, Bruining HA, Puppels GJ (2001). *In vivo* confocal Raman microspectroscopy of the skin: Noninvasive determination of molecular concentration profiles. Journal of Investigative Dermatology.

[27] Paquin R, Colomban P (2007). Nanomechanics of single keratin fibres: A Raman study of the α-helix → β-sheet transition and the effect of water. Journal of Raman Spectroscopy.

[28] Notingher I (2004). Discrimination between ricin and sulphur mustard toxicity *in vitro* using Raman spectroscopy. Journal of the Royal Society, Interface.

[29] Katainen E (2007). Quantification of the amphetamine content in seized street samples by Raman spectroscopy. Journal of forensic sciences.

[30] Fasman GD, Itoh K, Liu CS, Lord RC (1978). Laser-excited raman spectroscopy of biomolecules. XII. Thermally induced conformational changes in poly(L-glutamic acid). Biopolymers.

[31] Frushour BG, Koenig JL (1975). Raman studies of the crystalline, solution, and alkaline-denatured states of beta-lactoglobulin. Biopolymers.

[32] Zhang G, Moore DJ, Flach CR, Mendelsohn R (2007). Vibrational microscopy and imaging of skin: from single cells to intact tissue. Anal Bioanal Chem.

[33] Choe, C.-S., Lademann, J. & Darvin, M. E. Gaussian-function-based deconvolution method to determine the penetration ability of petrolatum oil into *in vivo* human skin using confocal Raman microscopy. *Laser Physics* **24**, 10.1088/1054-660x/24/10/105601 (2014).

[34] Movasaghi Z, Rehman S, Rehman IU (2007). Raman Spectroscopy of Biological Tissues. Applied Spectroscopy Reviews.

[35] Lefevre T, Rousseau ME, Pezolet M (2007). Protein secondary structure and orientation in silk as revealed by Raman spectromicroscopy. Biophys J.

[36] Yager, P. In *Biological applications of* Raman *spectroscopy* (ed. Spiro,T. G.) 203–261 (Wiley, 1987).

[37] Lord RC, Yu NT (1970). Laser-excited Raman spectroscopy of biomolecules. I. Native lysozyme and its constituent amino acids. Journal of molecular biology.

[38] Lord RC, Yu NT (1970). Laser-excited Raman spectroscopy of biomolecules. II. Native ribonuclease and alpha-chymotrypsin. Journal of molecular biology.

[39] Krafft C, Neudert L, Simat T, Salzer R (2005). Near infrared Raman spectra of human brain lipids. *Spectrochimica acta*. Part A, Molecular and biomolecular spectroscopy.

[40] Siamwiza MN (1975). Interpretation of the doublet at 850 and 830 cm^−1^ in the Raman spectra of tyrosyl residues in proteins and certain model compounds. Biochemistry.

[41] Verma SP, Wallach DFH (1977). Changes of Raman-scattering in CH-stretching regions during thermally induced unfolding of Ribonuclease. Biochemical and biophysical research communications.

[42] Gniadecka M (1998). Water and protein structure in photoaged and chronically aged skin. The Journal of investigative dermatology.

[43] Choe C, Lademann J, Darvin ME (2016). Lipid organization and stratum corneum thickness determined *in vivo* in human skin analyzing lipid–keratin peak (2820–3030 cm^−1^) using confocal Raman microscopy. Journal of Raman Spectroscopy.

[44] Nakazawa H, Ohta N, Hatta I (2012). A possible regulation mechanism of water content in human stratum corneum via intercellular lipid matrix. Chem Phys Lipids.

[45] Caussin J (2007). Lipophilic and hydrophilic moisturizers show different actions on human skin as revealed by cryo scanning electron microscopy. Exp Dermatol.

[46] Choe C, Lademann J, Darvin ME (2015). Analysis of Human and Porcine Skin *in vivo*/*ex vivo* for Penetration of Selected Oils by Confocal Raman Microscopy. Skin pharmacology and physiology.

[47] Imokawa G, Kuno H, Kawai M (1991). Stratum corneum lipids serve as a bound-water modulator. The Journal of investigative dermatology.

[48] Yamamura T, Tezuka T (1989). The water-holding capacity of the stratum corneum measured by 1H-NMR. The Journal of investigative dermatology.

[49] Yadav S, Pinto NG, Kasting GB (2007). Thermodynamics of water interaction with human stratum corneum I: measurement by isothermal calorimetry. Journal of pharmaceutical sciences.

[50] Rawlings AV (2014). Molecular basis for stratum corneum maturation and moisturization. The British journal of dermatology.

[51] Gilard V (1998). Measurement of total water and bound water contents in human stratum corneum by *in vitro* proton nuclear magnetic resonance spectroscopy. International Journal of Cosmetic Science.

[52] Visscher MO, Tolia GT, Wickett RR, Hoath SB (2003). Effect of soaking and natural moisturizing factor on stratum corneum water-handling properties. Journal of cosmetic science.

[53] Akhalaya MY, Maksimov GV, Rubin AB, Lademann J, Darvin ME (2014). Molecular action mechanisms of solar infrared radiation and heat on human skin. Ageing research reviews.

[54] Darvin ME (2007). *In vivo* Raman spectroscopic analysis of the influence of IR radiation on the carotenoid antioxidant substances beta-carotene and lycopene in the human skin. Formation of free radicals. Laser Phys. Lett..

[55] Robert C, Bonnet M, Marques S, Numa M, Doucet O (2015). Low to moderate doses of infrared A irradiation impair extracellular matrix homeostasis of the skin and contribute to skin photodamage. Skin pharmacology and physiology.

[56] Schleusener J, Lademann J, Darvin ME (2017). Depth-dependent autofluorescence photobleaching using 325, 473, 633, and 785 nm of porcine ear skin *ex vivo*. Journal of biomedical optics.

[57] Darvin ME, Meinke MC, Sterry W, Lademann J (2013). Optical methods for noninvasive determination of carotenoids in human and animal skin. Journal of biomedical optics.

[58] Bielfeldt S (2009). Assessment of human stratum corneum thickness and its barrier properties by *in-vivo* confocal Raman spectroscopy. International Journal of Cosmetic Science.

[59] Crowther JM (2008). Measuring the effects of topical moisturizers on changes in stratum corneum thickness, water gradients and hydration *in vivo*. British Journal of Dermatology.

[60] Mujica Ascencio S (2016). Confocal Raman microscopy and multivariate statistical analysis for determination of different penetration abilities of caffeine and propylene glycol applied simultaneously in a mixture on porcine skin *ex vivo*. European journal of pharmaceutics and biopharmaceutics.

[61] Pudney PD (2012). A new *in vivo* Raman probe for enhanced applicability to the body. Applied spectroscopy.

[62] Wu, C.-F. *Experiments: planning, analysis, and parameter design optimization/C.F. Jeff Wu, Michael Hamada*. 630 (2000).

[63] Jarque CM, Bera AK (1987). A Test for Normality of Observations and Regression Residuals. International Statistical Review/Revue Internationale de Statistique.

[64] Darvin ME (2004). Noninvasive detection of beta-carotene and lycopene in human skin using Raman spectroscopy. Laser Physics.

